# Confirmation of the physiological principles of the sequential laser technique for twin–twin transfusion syndrome: A Sequential Trial subanalysis

**DOI:** 10.1002/pmf2.70100

**Published:** 2025-09-08

**Authors:** Tucker E. Doiron, Lisa M. Korst, Arlyn S. Llanes, Martha A. Monson, Andrew H. Chon, Giancarlo Mari, Ramen H. Chmait

**Affiliations:** ^1^ Division of Maternal‐Fetal Medicine, Department of Obstetrics and Gynecology, Keck School of Medicine University of Southern California Los Angeles California USA; ^2^ Division of Maternal‐Fetal Medicine, Department of Obstetrics and Gynecology Saint Louis University Missouri USA; ^3^ Childbirth Research Associates LLC North Hollywood California USA; ^4^ Division of Maternal‐Fetal Medicine, Department of Obstetrics and Gynecology University of Utah Health Salt Lake City Utah USA; ^5^ Division of Maternal‐Fetal Medicine, Department of Obstetrics and Gynecology Intermountain Healthcare Salt Lake City Utah USA; ^6^ Division of Maternal‐Fetal Medicine, Department of Obstetrics and Gynecology Oregon Health & Science University Portland Oregon USA; ^7^ Division of Maternal Fetal Medicine, Department of Obstetrics and Gynecology, Texas Children's Hospital Baylor College of Medicine Houston Texas USA

**Keywords:** fetal anemia, fetal surgery, laser photocoagulation of communicating vessels, laser surgery, middle cerebral artery peak systolic velocity, monochorionic twins, multifetal pregnancy

## Abstract

**Introduction:**

Twin–twin transfusion syndrome (TTTS) is a complication of monochorionic diamniotic twin pregnancies. It is commonly treated with laser surgery to separate the placental circulations. Perioperative changes in the fetal middle cerebral artery peak systolic velocity (MCA PSV) may characterize the intraoperative blood shifts that occur between the twins during surgery. This study compared changes in the donor MCA PSV for the “selective” versus the “sequential” laser surgery techniques.

**Methods:**

This is a post hoc analysis of the Sequential Trial, a randomized controlled trial that compared the two laser techniques. The distinguishing characteristic of the sequential technique is that arteriovenous anastomoses are ablated in an order that should allow for an intraoperative blood transfer from the hypervolemic recipient to the hypovolemic donor. We compared the difference (Delta) in donor MCA PSV multiples of the median (MoM) (24‐h postoperative minus preoperative value) by surgical technique. Because anemia is associated with a higher MCA PSV, a negative Delta is consistent with a drop in the MCA PSV and a gain in blood volume.

**Results:**

Of the 642 patients, 606 (94%) had data available for analysis; 30 (4%) of the donors died within 24 h of surgery, and 6 were missing data and excluded from the analysis. The mean Delta MCA PSV MoM for the donor twin was −0.053 (*p *< 0.0001) for the sequential and 0.001 (*p *> 0.999) for the selective techniques (*p *= 0.003 for comparison of techniques). Based on the presence versus absence of donor critical abnormal Doppler (CAD) parameters and arterioarterial anastomoses (AA), four risk categories for donor survival were described in the Sequential Trial. Mean Deltas for the sequential versus selective procedures in these four categories were as follows: Category 1, no CAD no AA −0.021 versus 0.000, *p *= 0.162; Category 2, CAD no AA −0.199 versus −0.034, *p *< 0.001; Category 3, no CAD with AA −0.049 versus 0.121, *p *= 0.010; and Category 4, CAD with AA 0.073 versus −0.040, *p *= 0.239. Linear regression for Delta yielded a coefficient for the sequential versus selective techniques of −0.053 (0.024 SE), *p *= 0.027.

**Conclusions:**

Patients receiving the sequential technique had a greater drop in the mean donor MCA PSV MoM, suggesting that the sequential technique facilitated an intraoperative net blood transfusion to the donor. This benefit was apparent in Categories 2 and 3.

## 1 INTRODUCTION

Twin–twin transfusion syndrome (TTTS) complicates 8%–10% of monochorionic diamniotic (MCDA) twin pregnancies [1]. While all MCDA pregnancies have vascular connections between the circulatory systems of the fetuses, not all MCDA pregnancies are complicated by TTTS [1]. It has been proposed that TTTS develops when there is an imbalance in blood flow between the fetuses, resulting in a donor and a recipient twin [1].

Perinatal mortality for untreated TTTS is high [2], making it paramount for both clinicians and their patients to optimize treatment options. Laser photocoagulation is currently the principal treatment for pregnancies complicated by TTTS [3, 4, 5]. In the fetoscopic selective laser photocoagulation (selective) technique, the vascular communications between the donor and recipient twins are occluded in no particular order [6]. The selective technique is currently one of the most widely used surgical treatments for TTTS [7, 8]. The sequential selective laser photocoagulation of communicating vessels (sequential) technique is a modification of the selective approach [9]. In this approach, the arteriovenous communications (AV) with unidirectional blood flow from the donor to the recipient twin are laser‐occluded first, followed by occlusion of AV anastomoses from the recipient to the donor [9]. This should allow for a net intraoperative blood transfer from the hypervolemic recipient to the hypovolemic donor [10].

The Sequential Trial [11] compared survival outcomes of the two techniques. In the primary analysis, there were no differences in donor twin survival after selective versus sequential laser surgery. Based on post‐hoc analysis done and published in the original RCT [11]. suggested that the laser surgery technique (selective vs sequential) may need to be adapted based on the presence of two key risk factors: any preoperative donor twin critically abnormal Doppler (CAD) parameters (i.e., umbilical artery persistent absent/reversed end‐diastolic flow [UA A/REDF], ductus venosus [DV] persistent absent/reversed a‐wave, or middle cerebral artery peak systolic velocity [MCA PSV] multiples of the median [MoM] ≥1.5), and the intraoperative finding of arterioarterial anastomoses (AA).

**FIGURE 1:**
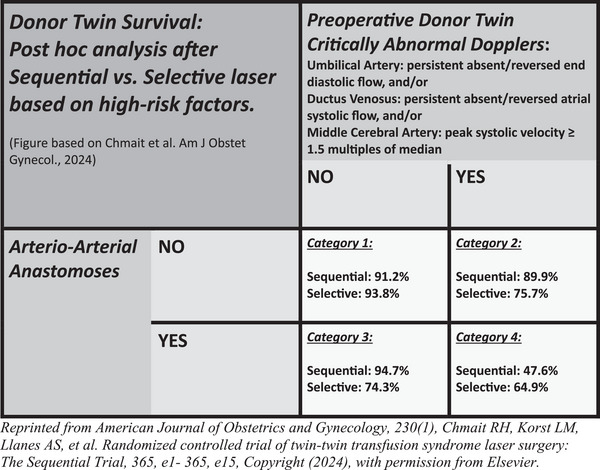
Donor twin survival by laser technique and risk category. The proportion of the Sequential Trial patients with donor twin survival in each of four categories (based on critically abnormal Doppler [CAD] parameters and the presence of arterioarterial anastomoses [AA]) by sequential versus selective surgical techniques. The categories are as follows: Category 1, no CAD and no AA; Category 2, CAD with no AA; Category 3, no CAD with AA; and Category 4, CAD with AA.

Figure 1 depicts the assignation of four categories that were studied in this post hoc analysis: Category 1, no CAD and no AA (similar donor twin survival by laser technique); Category 2, CAD and no AA (improved donor survival with sequential technique); Category 3, no CAD with AA (improved donor survival with sequential technique); and Category 4, CAD with AA (similar donor twin survival by laser technique) [11].

Thus, in the post hoc analyses from the Sequential Trial, donor twins in Categories 2 and 3 showed improved survival with the sequential technique, and similar donor twin survival for either laser technique for Category 1 patients. This observation raises the hypothesis that the intraoperative blood transfer that is theoretically associated with the sequential technique may benefit patients in Categories 2 and 3, and cause no harm in Category 1. However, Category 4 patients seemed to have worse donor twin survival after sequential laser surgery, the etiology of which remains unknown.

The measurement of the MCA PSV is typically used preoperatively to assess for fetal anemia [12]. A high MCA PSV MoM (e.g., ≥ 1.5) is considered to be a marker for fetal anemia, and in general, higher values are suggestive of a lower fetal hematocrit [12]. Furthermore, an elevated postoperative MCA PSV in TTTS has also been associated with neurodevelopmental impairment in the donor twin [13].

In this study, we used the difference in the donor MCA PSV MoM (postoperative minus preoperative value) to test for evidence of a change in hematocrit in the sequential versus the selective laser techniques, hypothesizing that a negative difference (i.e., the postoperative value is less than preoperative value) would indicate a drop in the MCA PSV MoM and an increase in the hematocrit of the volume‐depleted donor twin. This would support the rationale for using the sequential technique, particularly for those patients in Categories 2 and 3. Thus, the aims of this study were twofold: first, to determine whether our findings show a physiological difference between the two techniques by risk category; and second, to evaluate the potential for a customized operative approach to enhance donor survival.

## 2 MATERIALS AND METHODS

This is a post hoc analysis of data from the Sequential Trial, an open‐label randomized controlled trial that compared outcomes with sequential and selective laser techniques for TTTS patients. This trial was registered with ClinicalTrials.gov (identification number NCT02122328). Details on trial design, randomization, surgical technique, and findings have been previously reported [11]. The study was approved by the Institutional Review Board (IRB) for the University of Southern California (USC) Keck School of Medicine Health Sciences Campus.

The original study population consisted of all patients diagnosed with MCDA multiple gestations complicated by TTTS who underwent fetoscopic selective laser photocoagulation at a gestational age (GA) between 16 and 26 weeks at a single fetal surgery center from June 10, 2010 through November 10, 2022 and were enrolled in the Sequential Trial. TTTS was diagnosed if the donor twin had a maximum vertical pocket (MVP) ≤ 2 cm and the recipient twin had an MVP ≥ 8 cm on ultrasonography. Severity was classified using the Quintero staging system [14]. Patients were counseled on management options including (1) expectant management, (2) pregnancy termination, (3) amnioreduction, (4) fetoscopic laser surgery, and (5) umbilical cord occlusion. Patients were randomly assigned to the sequential versus the selective procedure.

This post hoc analysis of the original RCT evaluated changes in donor MCA PSV following laser photocoagulation. The patients were subclassified by laser type (selective vs. sequential) and by the risk categories described above. Procedure assignments from the original RCT were maintained, consistent with the original intention‐to‐treat design [15]. Of those assigned to the sequential group, 7.8% had the selective (standard) procedure, while 10.9% of those assigned to the standard procedure had the sequential or no procedure [11]. Each patient underwent a preoperative evaluation and ultrasound including Doppler assessment of the donor MCA PSV on the day prior to surgery. Those assigned to the selective technique had vascular anastomoses laser ablated in no particular order, while those assigned to the sequential technique had AV communications from the donor to the recipient (DR) ablated first, followed by those from the recipient to the donor (RD). The Solomon technique was not routinely used during the study period. The postoperative scan was completed on postoperative Day 1; approximately 24 h after surgery. If a donor MCA PSV value was missing, this patient was excluded from the analyses.

We compared the difference (Delta) in donor twin MCA PSV MoM (postoperative minus preoperative) by surgical technique (sequential vs. selective). Anemia is associated with a higher MCA PSV, so a negative Delta is consistent with an increased postoperative blood volume. The Delta MCA PSV was calculated for the donor twin based on procedure type (sequential vs. selective) and stratified according to the high‐risk categories described above.

Bivariate analyses between patient characteristics and the four risk categories were conducted. In stratified analyses, for each risk category, the donor MCA PSV MoM Delta was compared for the sequential versus selective procedures using the Wilcoxon rank sum test; for each procedure option, the distribution of the Delta was tested for its difference from zero using the Wilcoxon signed‐rank test. A second set of bivariate analyses was conducted to test for association between patient characteristics and the outcome, the donor MCA PSV MoM Delta value, to identify potential covariates for a multiple linear regression model. Predictors in this model included the independent variable, use of the sequential procedure, the risk categories (reference = Category 1), and patient characteristics found to be associated with the outcome in bivariate analyses (*p *< 0.10). All analyses were conducted per gestation and were performed in SAS v9.4 (Cary, NC). Continuous variables are reported as the mean ± standard deviation (SD) or median (range); group differences were compared using the Kruskal–Wallis test. Proportions were compared using the chi‐square test or the Fisher's exact test, as appropriate. Statistical significance was defined as *p *< 0.05. For multiple linear regression models, the regression parameters (β) are presented with the standard error (SE).

## 3 RESULTS

**FIGURE 2:**
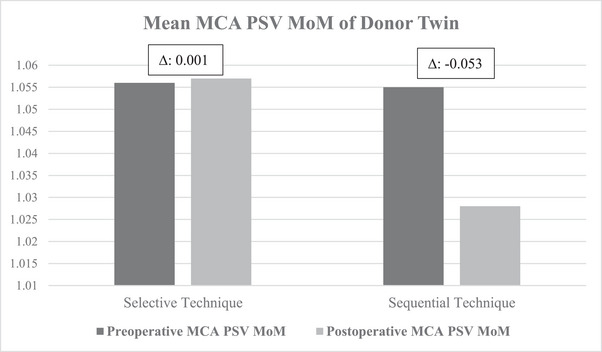
Mean MCA PSV MoM of the donor twin pre‐ and post‐laser surgery by technique (all categories).

Of the 642 patients in the original trial, 30 donors died within 24 hours of surgery prior to acquiring postoperative MCA PSV MoM, 5 were missing the postoperative MCA PSV MoM record, and 1 was missing the preoperative MCA PSV MoM record, yielding 606 study patients. Patients had the following distribution by risk category: Category 1 (no donor twin CAD and no placental AA anastomosis), *n* = 341, 56.3%; Category 2 (CAD with no AA), *n *= 133, 21.9%; Category 3 (no CAD with AA), *n* = 68, 11.2%; Category 4 (CAD with AA), *n* = 64, 10.6%. Baseline maternal, fetal, and neonatal characteristics of the participants are described by risk category in Table 1. Baseline Doppler characteristics of the donor twin by category are detailed in Table 2. For the 606 study patients, the mean (SD) Delta MCA PSV MoM for the donor twin was −0.053 (0.306) (*p *< 0.0001) for the sequential and 0.001 (0.286) (*p *> 0.999) for the selective techniques, with *p *= 0.003 for the technique comparison (Figure 2).

**TABLE 1:** Maternal, fetal, and neonatal characteristics of patients classified by risk category.^a^, ^b^

Characteristics	Total patients (*N* = 606)	Category 1 No CAD No AA (*N* = 341)	Category 2 CAD no AA (*N* = 133)	Category 3 No CAD +AA (*N* = 68)	Category 4 CAD + AA (*N* = 64)	*p* value
Age (years)	30.0 ± 5.9	30.1 ± 5.8	29.9 ± 5.7	30.6 ± 6.2	29.3 ± 6.0	0.542
Gestational age at surgery (weeks)	20.3 ± 2.4	20.6 ± 2.4	19.9 ± 2.4	20.8 ± 2.5	19.2 ± 1.9	<0.0001
Triplets	20 (3.3%)	11 (3.2%)	5 (3.8%)	1 (1.5%)	3 (4.7%)	0.755
Triplet chorionicity: Dichorionic‐ Diamniotic Dichorionic‐Triamniotic	*N* = 20 1 (5.0%) 19 (95.0%)	*N* = 11 0 (0%) 11 (100%)	*N* = 5 0 (0%) 5 (100%)	*N* = 1 0 (0%) 1 (100%)	*N* = 3 1 (33.3%) 2 (67.7%)	0.113
Quintero Stage						<0.0001
1	101 (16.7%)	60 (17.6%)	8 (6.0%)	31 (45.6%)	2 (3.1%)	
2	150 (24.8%)	116 (34.0%)	15 (11.3%)	16 (23.5%)	3 (4.7%)	
3	311 (51.3%)	140 (41.1%)	101 (75.9%)	15 (22.1%)	55 (85.9%)	
4	44 (7.3%)	25 (7.3%)	9 (6.8%)	6 (8.8%)	4 (6.3%)	
Sequential procedure performed	312 (51.5%)	165 (48.4%)	77 (57.9%)	37 (54.4%)	33 (51.6%)	0.292
Sequential procedure assigned	306 (50.5%)	168 (48.3%)	67 (50.4%)	37 (54.4%)	34 (53.1%)	0.849
Gestational age at delivery	32.9 ± 4.0	32.9 ± 3.9	32.9 ± 3.8	33.1 ± 3.9	32.6 ± 4.8	0.922
IUFD donor within 24 h	1 (0.2%)	1 (0.3%)	0 (0%)	0 (0%)	0 (0%)	0.855
IUFD recipient within 24 h	7 (1.2%)	6 (1.8%)	0 (0%)	0 (0%)	1 (1.6%)	0.317
Born alive donor	540 (89.1%)	319 (93.5%)	115 (86.5%)	62 (91.2%)	44 (68.8%)	<0.0001
Born alive recipient	572 (94.4%)	319 (93.5%)	127 (95.5%)	66 (97.1%)	60 (93.8%)	0.632
Twin number alive at birth						0.001
0	18 (3.0%)	9 (2.6%)	4 (3.0%)	1 (1.5%)	4 (6.3%)	
1	64 (10.6%)	26 (7.6%)	16 (12.0%)	6 (8.8%)	16 (25.0%)	
2	524 (86.5%)	306(89.7%)	113 (85.0%)	61 (89.7%)	44 (68.8%)	
Twin number alive at 30 days						<0.001
0	30 (5.0%)	14 (4.1%)	8 (6.0%)	3 (4.4%)	5 (7.8%)	
1	77 (12.7%)	33 (9.7%)	17 (12.8%)	8 (11.8%)	19 (29.7%)	
2	499 (82.3%)	294 (86.2%)	108 (81.2%)	57 (83.8%)	40 (62.5%)	
30‐day survival						
Donor	522 (86.1%)	311 (91.2%)	110 (82.7%)	59 (86.8%)	42 (65.6%)	<0.0001
Recipient	553 (91.3%)	310 (90.9%)	123 (92.5%)	63 (92.6%)	57 (89.1%)	0.837
Dual twin survival	499 (82.3%)	294 (86.2%)	108 (81.2%)	57 (83.8%)	40 (62.5%)	0.0001
At‐least‐one survivor	576 (95.0%)	327 (95.9%)	125 (94.0%)	65 (95.6%)	59 (92.2%)	0.574

Abbreviations: AA: arterioarterial anastomoses; CAD: critically abnormal Dopplers; IUFD: intrauterine fetal demise.

^a^ *N* (%) or mean ± standard deviation.

^b^ *p* value was calculated using chi‐square testing for categorical variables and Kruskal–Wallis testing for continuous variables.

**TABLE 2:** Donor twin pre‐ and postoperative Doppler findings^a^, ^b^ ^.^

Characteristics	Total patients (*N* = 606)	Category 1 No CAD No AA (*N* = 341)	Category 2 CAD no AA (*N* = 133)	Category 3 No CAD +AA (*N* = 68)	Category 4 CAD + AA (*N* = 64)	*p* value
Donor twin abnormal preoperative Doppler findings						
UA A/REDF	136 (22.4%)	0 (0%)	82 (61.7%)	0 (0%)	54 (84.4%)	NA
UV pulsatile flow	32 (5.3%)	8 (2.3%)	20 (15.0%)	1 (1.5%)	3 (4.7%)	<0.0001
DV absent/ reversed a‐wave	44 (7.3%)	0 (0%)	30 (22.6%)	0 (0%)	14(21.9%)	NA
MCA PSV MoM ≥1.5	55 (9.1%)	0 (0%)	44 (33.1%)	0 (0%)	11(17.2%)	NA
MCA PSV MoM ≥1.5 as the only indicator of CAD	42 (6.9%)	0 (0%)	34 (25.6%)	0 (0%)	8 (12.5%)	NA
MCA PSV MoM	1.05 ± 0.30	0.97 ± 0.21	1.24 ± 0.40	0.99 ± 0.18	1.16 ± 0.34	<0.0001
Postoperative Donor MCA PSV MoM	1.03 ± 0.30	0.96 ± 0.24	1.13 ± 0.36	1.02 ± 0.27	1.18 ± 0.38	<0.0001

Abbreviations: AA: arterioarterial anastomoses; CAD: critically abnormal Dopplers; DV, ductus venosus; MCA PSV MoM, middle cerebral artery peak systolic velocity multiples of the median; NA, not applicable; UA A/REDF, Umbilical artery absent/reversed end diastolic flow; UV: umbilical vein.

^a^ *N*(%) or mean ±  standard deviation.

^b^ *p* value was calculated using chi‐square testing for categorical variables and Kruskal–Wallis testing for continuous variables.

The overall mean (SD) GA at surgery was 20.3 (2.4) weeks; this varied by risk category (*p *< 0.0001) with those in Category 4 having the earliest mean GA of the procedure. The majority of patients (*N* = 417, 72.1%) delivered at ≥ 32 gestational weeks, with 115 (19.0%) delivering at a GA between 28 and < 32 weeks, 52 (8.9%) delivering between 24 and < 28 weeks, and 22 (3.6%) delivering before 24 weeks.

The only patient characteristic identified that was associated with the Delta MCA PSV MoM for the donor was Hispanic ethnicity, which had a Delta MCA PSV MoM of 0.029 ± 0.26 versus −0.042 ± 0.304 in non‐Hispanic patients (*p* < 0.008) (Table 3).

**TABLE 3:** Delta MCA PSV MoM (preoperative minus postoperative value) of the donor twin by patient characteristic.^a^, ^b^

	Delta MCA PSV MoM of the donor twin		
Patient characteristic	Patient characteristic present	Patient characteristic not present	*p* value
Hispanic	0.029 ± 0.267	−0.042 ± 0.304	0.008
IUGR donor	−0.028 ± 0.308	−0.024 ± 0.282	0.898
Quintero stage 3	−0.031 ± 0.307	−0.021 ± 0.288	0.447
Sequential procedure assigned	−0.053 ± 0.306	0.001 ± 0.286	0.003
Placenta anterior	−0.046 ± 0.299	−0.008 ± 0.296	0.147
Triplet gestation	0.047 ± 0.359	−0.029 ± 0.295	0.491
GA at surgery ≥20 weeks	−0.025 ± 0.304	−0.028 ± 0.291	0.951
Born alive donor	−0.035 ± 0.283	0.047 ± 0.391	0.130
Born alive recipient	−0.021 ± 0.299	−0.111 ± 0.263	0.150
GA at delivery ≥32 weeks	−0.031 ± 0.279	−0.018 ± 0.336	0.513

Abbreviations: GA: gestational age; IUGR, intrauterine growth restriction; MCA PSV MoM, middle cerebral artery peak systolic velocity multiples of the median.

^a^ mean ± standard deviation.

^b^ *p* value was calculated using Wilcoxon rank‐sum testing.

Each of the four risk categories was then analyzed separately, comparing the donor Delta MCA PSV MoM for the sequential and selective procedures (Table 4). The following findings were noted:

- Category 1 (no CAD no AA) (*n* = 341) −0.021 ± 0.275 versus 0.000 ± 0.244, *p *= 0.162
- Category 2 (CAD no AA) (*n* = 133) −0.199 ± 0.333 versus −0.034 ± 0.322, *p *< 0.001
- Category 3 (no CAD with AA) (*n* = 68) −0.049 ± 0.262 versus 0.121 ± 0.305, *p *= 0.010
- Category 4 (CAD with AA) 0.073 ± 0.353 versus −0.040 ± 0.380, *p *= 0.239

**TABLE 4:** Delta MCA PSV MoM of donor twin (postoperative minus preoperative value) by assigned laser technique and risk category.^a^

Patient characteristic	Delta MCA PSV MoM^b^	*p* value^c^, ^d^
Category 1 (no CAD no AA)		
All patients (*N* = 341)	−0.010 ± 0.259	0.080
Sequential procedure assigned (*N* = 168)	−0.021 ± 0.275	0.036
Selective procedure assigned (*N* = 173)	0.000 ± 0.244	0.687
*p* value difference in procedure	0.162	
Category 2 (CAD no AA)		
All patients (*N* = 133)	−0.117 ± 0.337	<0.0001
Sequential procedure assigned (*N* = 77)	−0.199 ± 0.333	<0.0001
Selective procedure assigned (*N* = 66)	−0.034 ± 0.322	0.580
*p* value difference in procedure	<0.001	
Category 3 (no CAD with AA)		
All patients (*N* = 68)	0.028 ± 0.293	0.445
Sequential procedure assigned (*N* = 37)	−0.049 ± 0.262	0.210
Selective procedure assigned (*N* = 31)	0.121 ± 0.305	0.019
*p* value difference in procedure	0.010	
Category 4 (CAD with AA)		
All patients (*N* = 64)	0.020 ± 0.367	0.675
Sequential procedure assigned (*N* = 34)	0.073 ± 0.353	0.294
Selective procedure assigned (*N* = 30)	−0.040 ± 0.380	0.561
*p* value difference in procedure	0.239	

Abbreviations: AA, arterioarterial anastomosis; CAD, critically abnormal Dopplers.

^a^ mean ± standard deviation.

^b^ MCA PSV MoM: middle cerebral artery peak systolic velocity multiples of the median.

^c^ *p* value for the Delta MCA PSV MoM difference from zero was calculated using Wilcoxon signed‐rank testing.

^d^ *p* value for the Delta MCA PSV MoM difference by procedure was calculated using Wilcoxon rank‐sum testing.

The multiple linear regression model for the effect of the sequential technique on Delta MCA PSV MoM, controlling for the risk categories and Hispanic ethnicity, yielded a regression parameter for the sequential versus selective techniques of −0.053 (0.024 SE), *p *= 0.027 (Table 5). When patients in Category 4 were eliminated from the denominator, this resulted in a regression parameter of −0.072 ± 0.024, *p *= 0.003.

**TABLE 5:** Multiple linear regression model for Delta MCA PSV MoM by procedure.^a^

Patient characteristic	Regression parameter (β) + standard error	*p* value
Sequential procedure assigned (reference = selective procedure assigned)	−0.053 ± 0.024	0.027
Risk category (reference = Category 1 (no CAD no AA)		
Category 2 (CAD no AA)	−0.106 ± 0.030	<.001
Category 3 (No CAD with AA)	0.033 ± 0.039	.399
Category 4 (CAD with AA)	0.027 ± 0.040	.492
Hispanic ethnicity	0.063 ± 0.029	.028

Abbreviations: AA, arterioarterial anastomosis; CAD, critically abnormal Dopplers.

^a^ mean ± standard deviation.

In the first 24 h after surgery, 4% of donors died. These donors were excluded from our analysis. Following the first 24 h after surgery, donor demise occurred in 11% of remaining patients, resulting in a total donor fetal death rate of 15%. An additional 14% of donor twins that were born alive died within the first 30 days of life.

## 4 DISCUSSION

When analyzing all patients together, the donor Delta MCA PSV MoM exhibited a more significant decrease following the sequential laser technique compared to the selective (standard) method for TTTS treatment. When stratified by risk category, the sequential laser technique consistently resulted in a larger drop in donor Delta MCA PSV MoM in Categories 2 and 3, those with donor twin CAD or the presence of placental arterioarterial anastomosis (AA), but not both. The multiple linear regression model estimated an overall Delta (SD) of −0.053 ± 0.024 for the sequential procedure, and increased to −0.072 ± 0.024 when patients in Category 4 were excluded.

The decrease in donor MCA PSV MoM after laser surgery in those receiving the sequential technique was apparent in risk Categories 2 and 3, while an increase was noted in Category 3 (No CAD with AA) for those receiving the selective technique. These findings suggest that donor twins in Categories 2 and 3 may have improved fetal hemoglobin levels after the sequential laser technique. This aligns with previous research on red cell alloimmunization, which has shown an inverse correlation between MCA PSV and fetal hemoglobin levels [16], as well as the immediate decrease in MCA PSV following anemia correction in fetuses undergoing intravascular transfusion [17]. Consequently, our results indicate that the sequential technique may restore hemoglobin levels in the donor twin more rapidly than with the selective approach, potentially benefiting patients in Categories 2 and 3. Interestingly, in the Sequential Trial, there appeared to be improved survival of the donor twin in Categories 2 and 3, the very group that had the significant improvement in the MCA PSV MoM. Further study will be required to understand the clinical implications of these findings.

The original analysis in the Sequential Trial revealed no apparent association between overall donor survival with the sequential versus selective laser technique (85.6% vs. 84.2%) [11]. On further investigation of this cohort post hoc, there appeared to be differences in donor survival noted with selective versus sequential techniques based on the presence or absence of both donor twin preoperative CAD and the presence of an AA placental anastomosis. These predisposing factors have been shown to place the donor twin at higher risk for demise postoperatively [11, 18]. In the Sequential Trial, donor twin survival appeared to be improved with the sequential technique in those pregnancies with either CAD or AA present but not both, which is the case in Category 4 (Figure 1). This group included the most severely affected donor fetuses, and several factors may account for this lack of response. First, these fetuses may have had mild or absent anemia, accounting for the absence of observed changes. Alternatively, they may have been severely anemic, and unable to tolerate transfusion, as seen in cases of severe anemia due to red cell alloimmunization, where fetuses may die within 24 h due to volume overload. In such cases, it is crucial to transfuse severely anemic fetuses gradually to increase hematocrit over multiple procedures [19]. Lastly, the small number of fetuses in this category might have limited the ability to detect any changes; a larger sample size could potentially reveal different outcomes. Given these considerations, further investigation is necessary to understand why patients in this group responded differently to the sequential laser technique compared to other Categories.

Neurological outcome after laser‐treated TTTS is an important consideration given the proposed perioperative hemodynamic shifts, and the MCA PSV may give further insight into the impacts these shifts have on neurodevelopment of the fetus. While some studies have shown that survivors of laser‐treated TTTS have an incidence of neurodevelopmental impairment (NDI) of approximately 4%–10% [3], another reported that overall cognitive quotients were within the normal range for survivors at 2 years of age [20]. The etiology of neurodevelopmental impairment after laser surgery is suspected to be due to perioperative hemodynamic shifts during laser ablation, as previously mentioned [20, 21]. More specifically, it has been proposed that a postoperative increase in the MCA PSV or umbilical artery pulsatility index is associated with a higher incidence of NDI [13]. In our analysis, the sequential procedure was associated with a larger intraoperative blood transfer to the donor twin, resulting in a decreased postoperative MCA PSV, which we hypothesize may be protective with respect to the occurrence of NDI of the donor twin. Further study will be required to determine if the sequential procedure is clinically neuroprotective to the donor twin over the selective technique.

This study has several strengths. This is a post hoc analysis of the largest RCT to date evaluating treatment of TTTS; this large sample size allowed for categorization into 4 groups to evaluate distinct donor survival patterns in each group. Given that all cases were done at a single surgery center, there was consistency with surgical technique and setup, and uniform ultrasound measurements of MCA PSV. Survivorship and outcomes of all studied cases was known as no cases were lost to follow‐up.

There are several limitations to this study. The unplanned nature of the post hoc analysis weakens the interpretation of the findings; however, such analyses can be helpful for generating hypotheses for future prospective trials. Furthermore, the sample size for those in Category 4 was small, limiting the interpretation of the results in a group with the highest risk of poor outcomes. Average operative time between the techniques was not significant; however, we do not have data on time interval between laser photocoagulation of the donor‐to‐recipient anastomoses and recipient‐to‐donor anastomoses, which may play a role in the amount of hemodynamic shift that occurs between twins. Furthermore, this study is a retrospective evaluation of a study population; therefore, there is no information on long‐term outcomes.

## 5 CONCLUSION

Overall, the Delta MCA PSV of the donor suggested a net intraoperative blood shift to the donor twin with the sequential versus the selective technique in risk Categories 2 and 3, those with either CAD or AA, but not both. Categories 2 and 3 were also the two groups that appeared to have improvement in donor twin survival post‐sequential laser surgery. These findings corroborate the physiological principles of the sequential laser technique and provide further insight for customization of surgical technique based on the presence or absence of CAD and AA.

## CONFLICT OF INTEREST STATEMENT

L.M.K. is an independent contractor who assists with research studies. The remaining authors declare no conflicts of interest.
